# The Rare Occurrence of Post-tonsillectomy Surgical Emphysema

**DOI:** 10.7759/cureus.16430

**Published:** 2021-07-16

**Authors:** Hazim M Aleid, Danah F Alrusayyis, Aishah A AlGhuneem

**Affiliations:** 1 Pediatric Otorhinolaryngologist, King Fahad Specialist Hospital, Dammam, SAU; 2 College of Medicine, Imam Abdulrahman Bin Faisal University, Dammam, SAU

**Keywords:** tonsillectomy, post-tonsillectomy complication, surgical emphysema, gag-reflex, facial emphysema

## Abstract

Subcutaneous facial emphysema is a rare complication of tonsillectomy that can lead to infection, upper airway obstruction or invasion into the thorax. The latter can cause pneumomediastinum or pneumothorax, with possible subsequent cardiorespiratory function impairment. Although multiple causes are suggested in the literature, the main causative factor is still unclear. Moreover, the rationale for its management is inconsistent and the outcomes are unpredictable. We report a case of a 14-year-old pediatric male patient, known to have a hypersensitive gag reflex, who developed post-tonsillectomy cervicofacial subcutaneous emphysema; management has achieved complete clinical resolution after two weeks of complication onset. Additionally, we present a literature review that showcases the potential causes and management of subcutaneous emphysema.

## Introduction

Tonsillectomy is a safe, routine surgical procedure in the otorhinolaryngology specialty [1]. According to a large retrospective analysis, the rate of occurrence of any complication within 30 days after tonsillectomy is 21.46%. Post-operative infection (16.1%), pain (13.1%) and bleeding (6.37%) were the most commonly encountered complications [2]. Others include nausea, vomiting, lingual edema with or without airway obstruction and injury to the glossopharyngeal nerve or carotid artery [1,3,4]. Subcutaneous facial emphysema is an additional rare, life-threatening complication that cannot be overlooked. It is defined by the presence of air between the fascial planes of the connective tissue and can lead to infection, upper airway obstruction and invasion into the thorax. The latter can cause pneumomediastinum or pneumothorax, with possible subsequent cardiorespiratory function impairment [3,4]. According to the literature, the first case of cervicofacial subcutaneous emphysema was diagnosed in 1936 [5]. Another study concluded that only 27 cases were reported in the period between 2000 and 2020 [6]. Although multiple theories are suggested in the literature, the main causative factor is still unclear [1]. We report a case of a 14-year-old pediatric male patient, known to have a hypersensitive gag reflex, who developed post-tonsillectomy cervicofacial subcutaneous emphysema. Adding to the rarity of the complications, the clinical presentation suggests different theories of causation that will be further discussed.

## Case presentation

A 14-year-old Saudi male was admitted for an elective tonsillectomy to treat recurrent tonsillitis. He had poor oral intake secondary to severe vomiting and hypersensitive gag reflex that necessitated frequent admissions in his previous episodes. His medical, neurological, surgical and family history are otherwise irrelevant. Examination revealed a grade 2 tonsillar enlargement with a normal-looking soft palate. The patient's BMI and pre-operative assessments were all within the normal range.

Under a standardized protocol, anesthesia and uneventful intubation were performed by an expert anesthesiologist. Tonsillectomy was performed by needle cautery whereas suction cautery was used for the superior and inferior poles of the tonsils. A 21-gauge needle was used to inject 2 ml of Marcaine into the tonsillar pillars. No peritonsillar bed invasion or injury was observed.

**Figure 1:**
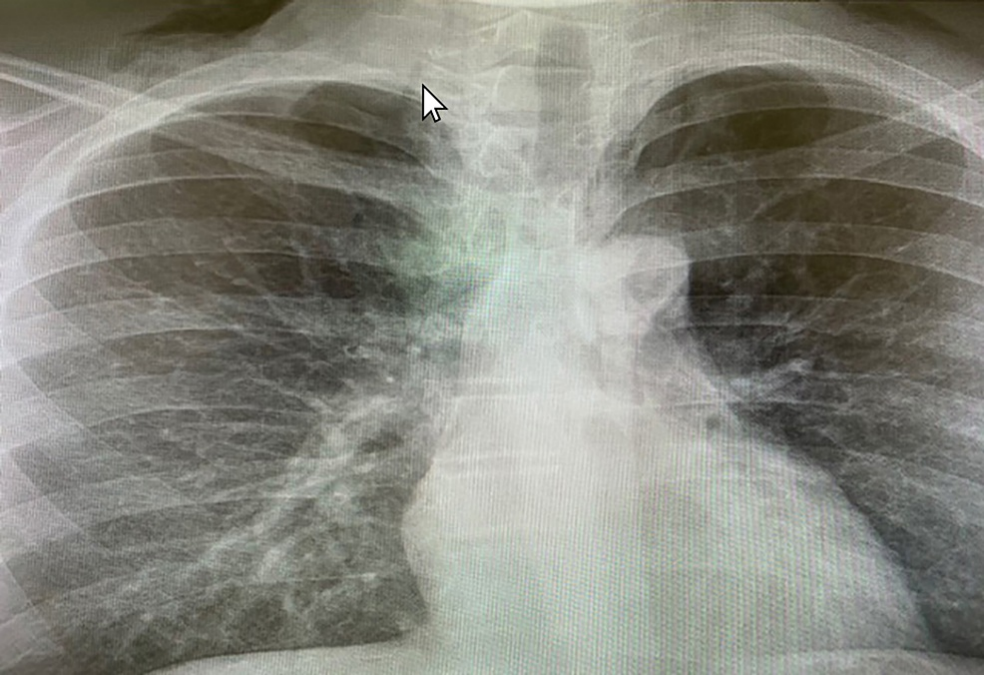
Plain X-ray of a posteroanterior view showing a bilateral lower neck and soft tissue subcutaneous emphysema. The arrow points to a gaseous streak seen centrally at the root of the neck.

**Figure 2:**
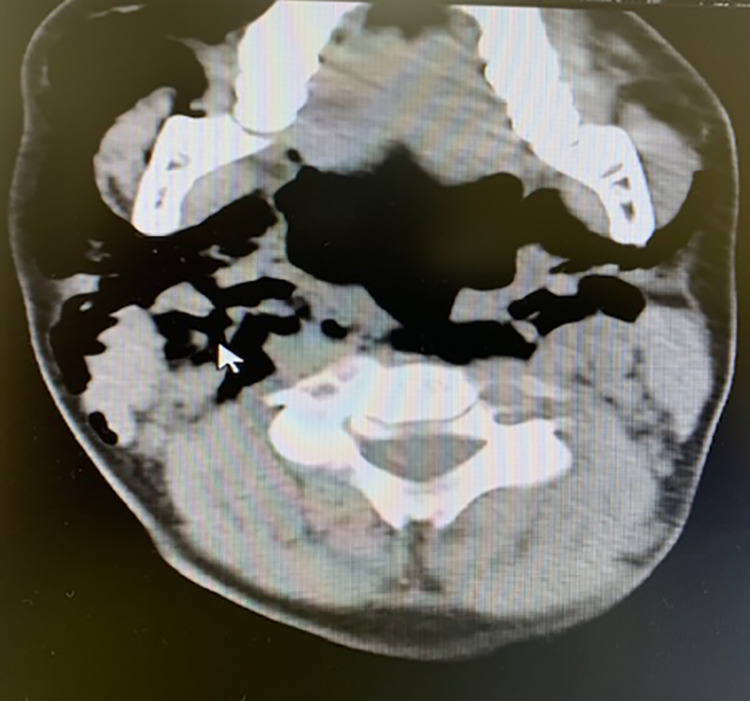
Non-contrast axial CT scan showing bilateral subcutaneous emphysema in the neck, more prominent in the right side, with mild pneumomediastinum.

In the recovery room, the patient had a sudden onset of right cheek and jaw swelling. He had a strong gag reflex after the resolution of anesthesia. Oral examination and oxygen saturation were normal with no signs of airway compromise. An immediate plain radiograph showed a bilateral lower neck subcutaneous emphysema with gaseous streaks seen centrally at the root of the neck (Figure 1). CT scan revealed bilateral subcutaneous emphysema in the neck, more prominent in the right side, with mild pneumomediastinum (Figure 2). There was no evidence of pneumothorax in plain radiograph nor CT scan.

**Figure 3:**
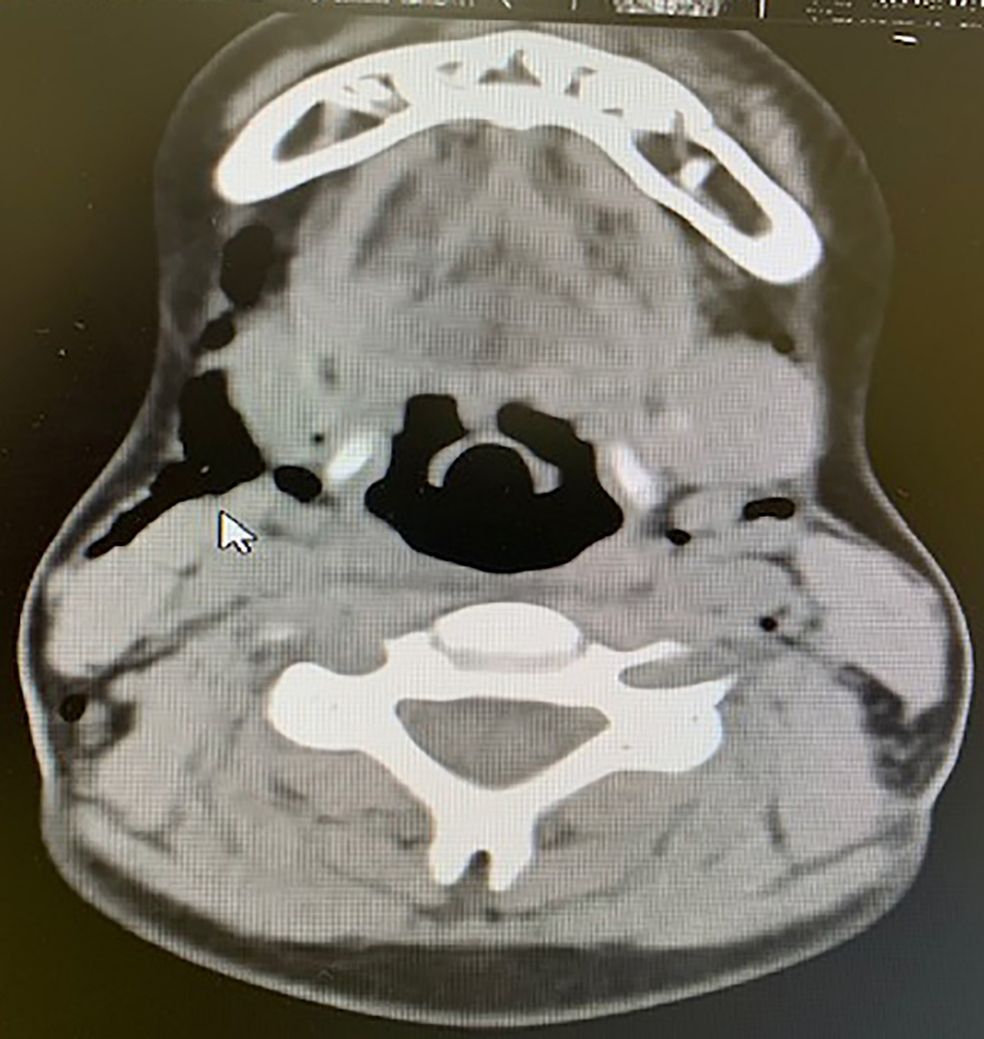
Non-contrast axial CT scan shows a significant regression of the emphysema on the fifth postoperative day.

The patient was admitted for observation. Broad-spectrum antibiotics were administered intravenously and oral intake was stringently restricted. He also received one trunk of oxygen therapy. Neither progression of emphysema nor signs of airway obstruction were documented. On the fifth postoperative day, a CT scan showed a significant regression of the emphysema (Figure 3). The patient was discharged after one week with marked improvement in oral intake. He was prescribed oral antibiotics and analgesics. After one week, an in-office examination revealed complete healing of the tonsillar bed and resolution of facial and mediastinal subcutaneous emphysema.

## Discussion

Subcutaneous emphysema is a rare but potentially life-threatening complication of tonsillectomy [6,7]. Recurrent tonsillitis and positive history of peritonsillar abscess predispose to fibrosis and adhesions of the tonsillar tissue and underlying muscular layers. This can increase the risk of subcutaneous emphysema [8]. In this report, the patient had a history of recurrent tonsillitis with poor oral intake secondary to severe vomiting and hypersensitive gag reflex.

Although there is no identified etiology, several theories suggested possible pathogeneses of this condition. The first and the most acknowledged theory is the descending mechanism. It proposes that the iatrogenic damage of the mucosa of the pharyngolaryngeal wall during surgery or anesthesia enables air to travel through the superior constrictor muscle into the masticator and pharyngeal spaces. As a consequence, coughing, straining and vomiting increase the upper airway pressure and ease the accumulation of air. This accumulation may extend to the mediastinum because of the anatomical connections between laryngeal and pharyngeal spaces [6,9-13]. The second theory is called the ascending mechanism. It suggests that an uncontrolled increase in intrapulmonary pressure ruptures marginal alveoli, causing pneumomediastinum that can extend further to the neck [5,8-11]. The third theory states that released gases by organisms and neighboring solutions can be liberated into enclosed spaces leading to emphysema [6].

Crepitus on examination can be a sign of subcutaneous emphysema and radiological imaging, such as plain radiograph and CT scan, can detect air presenting in subcutaneous spaces. If a patient with subcutaneous emphysema also developed dyspnea, dysphagia, chest and back pain, cyanosis and Hamman's sign (crepitus synchronous with systole), pneumomediastinum should be suspected [9,11,14]. In our case, CT scan supported the findings of the initial plain radiograph and revealed bilateral subcutaneous emphysema in the neck, more prominent in the right side, with mild pneumomediastinum. Moreover, the patient had a vaguely persistent strong gag reflex after the resolution of anesthesia. Oral examination and oxygen saturation were normal with no signs of airway compromise.

The treatment of subcutaneous emphysema varies according to its severity. Some cases resolve with observation alone [9,14,15]. However, associated infection, respiratory distress or severe pain necessitate hospitalization and oral restrictions. A regular assessment of the extent of emphysema and airway is needed [12,15]. Broad-spectrum antibiotics may also be prescribed [9,11,14]. Caution must be taken to treat pharyngeal and tonsillar mucosal damage [9,12,13]. The injured mucosa may be sutured to prevent secondary entrance of bacteria and to limit the extension of surgical emphysema [11,14,15]. A cold compress can be applied to the site of the swelling. Moreover, if a patient is suffering from respiratory distress, 100% O2 should be administered [9,12-15]. The treating physician should anticipate tracheostomy at any moment in case severe progressive emphysema results in airway obstruction [9,13,14]. In our case, the patient was admitted for observation. Broad-spectrum antibiotics were administered intravenously with oral restrictions. He also received one trunk of oxygen therapy. There was no progression of emphysema or signs of airway obstruction, and the complete resolution was documented after two weeks of the onset of the complication. The patient was prescribed oral antibiotics and analgesics.

Activities that should be avoided include straining, vomiting and coughing as they increase upper airway pressure [8,10,11,14,15].

## Conclusions

Subcutaneous emphysema and pneumomediastinum are rare occurrences but potentially fatal complications that can result from a simple procedure such as tonsillectomy. Several theories suggested the pathogeneses of this condition but the actual cause is still unknown. Early recognition of risk factors, diagnosis of emphysema, monitoring and treatment are important aspects as the management of every patient is tailored to the severity of their condition.
